# Long-term impact of a real-world coordinated lifestyle promotion initiative in primary care: a quasi-experimental cross-sectional study

**DOI:** 10.1186/s12875-014-0201-x

**Published:** 2014-12-16

**Authors:** Kristin Thomas, Barbro Krevers, Preben Bendtsen

**Affiliations:** Department of Medicine and Health Sciences, Linköping University, Linköping, Sweden; Department of Medical Specialist and Department of Medical and Health Sciences, Linköping University, Motala, Sweden

**Keywords:** Healthy lifestyle promotion, Primary care, Implementation, Coordinated care, RE-AIM framework, Maintenance

## Abstract

**Background:**

Integration of lifestyle promotion in routine primary care has been suboptimal. Coordinated care models (e.g. screening, brief advice and referral to in-house specialized staff) could facilitate lifestyle promotion practice; they have been shown to increase the quality of services and reduce costs in other areas of care. This study evaluates the long-term impact of a coordinated lifestyle promotion intervention with a multidisciplinary team approach in a primary care setting.

**Methods:**

A quasi-experimental, cross-sectional design was used to compare three intervention centres using a coordinated care model and three control centres using a traditional model of lifestyle promotion care. Outcomes were inspired by using the RE-AIM framework: reach, the proportion of patients receiving lifestyle promotion; effectiveness, self-reported attitudes and competency among staff; adoption, proportion of staff reporting daily practice of lifestyle promotion and referral; and implementation, of the coordinated care model. The impact was investigated after 3 and 5 years. Data collection involved a patient questionnaire (intervention, *n* = 433–497; control, *n* = 455–497), a staff questionnaire (intervention, *n* = 77–76; control, *n* = 43–56) and structured interviews with managers (*n* = 8). The χ^2^ test or Fisher exact test with adjustment for clustering by centre was used for the analysis. Problem-driven content analysis was used to analyse the interview data.

**Results:**

The findings were consistent over time. Intervention centres did not show higher rates for reach of patients or adoption among staff at the 3- or 5-year follow-up. Some conceptual differences between intervention and control staff remained over time in that the intervention staff were more positive on two of eight effectiveness outcomes (one attitude and one competency item) compared with control staff. The Lifestyle team protocol, which included structural opportunities for coordinated care, was implemented at all intervention centres. Lifestyle teams were perceived to have an important role at the centres in driving the lifestyle promotion work forward and being a forum for knowledge exchange. However, resources to refer patients to specialized staff were used inconsistently.

**Conclusions:**

The Lifestyle teams may have offered opportunities for lifestyle promotion practice and contributed to enabling conditions at centre level but had limited impact on lifestyle promotion practices.

## Background

Lifestyle-related illness and disease such as cardiovascular disease, cancer and diabetes are among the leading causes of death worldwide [1,2]. Healthy lifestyles can prevent 80% of coronary heart disease, 90% of type 2 diabetes, and 30% of different forms of cancer [3]. Health care organizations are therefore encouraged to introduce healthy lifestyle promotion in routine practice; that is, the promotion of a healthy diet, tobacco cessation, moderate drinking and regular physical activity [4]. In Sweden and internationally, lifestyle promotion practice has been augmented by national policies and practice guidelines [5-10]. Primary care has been identified as the setting that can offer continuous and comprehensive lifestyle promotion to patients with health-risk behaviours [11,12]. In general, the effectiveness of single and multiple lifestyle interventions has been found to be reasonable in a recent systematic review [13]. In addition, both group and individual interventions of varying intensity have been shown to be effective in supporting a healthy lifestyle [14].

However, the integration of lifestyle promotion in routine primary care has not been optimal [15]. The rate of lifestyle promotion in routine primary care has been shown to vary from a few percent to about 30% of patients who receive lifestyle advice [15-20]. A study investigating video recordings of consultations in Dutch general practices between 1975 and 2008 showed that only 6–13% of consultations included lifestyle advice [17].

The introduction of new practices under real-world conditions has been found to be challenging [21]. Specific barriers to fully integrating lifestyle promotion in primary care have been shown to be intrapersonal (perceived effectiveness of interventions, attitudes and confidence), interpersonal (patient characteristics) and institutional (resources) [11,22-28]. Implementation interventions aim to promote change in clinical behaviour; audits, feedback, education and reminders have had minor effects [29,30]. One intervention that could facilitate lifestyle promotion practice, specifically to overcome institutional barriers, is coordinated care [31]. The aim of coordinated care is to improve access, efficiency and quality of care [32]. Coordinated lifestyle promotion in primary care could entail screening for patients with risky behaviours, delivering brief advice and referral to in-house specialized staff [33]. Approaches to coordinated care have typically involved multidisciplinary teams, care management and disease management [32,34]. Coordinated care has been shown to improve service continuity and collaboration, increase the quality of services and reduce costs in mental health and chronic care [32,35]. However, more research is needed to investigate coordinated care in the area of lifestyle promotion [31].

This article reports on a real-world coordinated care intervention that used a multidisciplinary team approach. Similar coordinated care models have been used successfully for diabetes care whereby patients with increased glucose levels are referred to specialist diabetic nurses [36]. Primary care in Sweden consists of physicians, dieticians, behavioural therapists and specialized nurses. The intervention aimed to utilize the existing multidisciplinary structure of Swedish primary care.

### Aim

To evaluate the long-term impact of a coordinated lifestyle promotion intervention that used a multidisciplinary team approach in a primary care setting.

## Methods

### Design of the study

A quasi-experimental, cross-sectional design compared two groups of primary care centres: three intervention centres that used a coordinated care model and three control centres that used a traditional model of lifestyle promotion care. The definition of the outcome variables was inspired by the RE-AIM framework [37,38]. RE-AIM is an acronym for reach (of target population), effectiveness (impact on key outcomes), adoption (among staff and settings), implementation (consistency of the intervention) and maintenance (long-term impact on individual and setting levels). Table 1 shows the original RE-AIM definitions and the definitions used in this study. The impact on outcomes was investigated at 3- and 5-year follow-ups; the primary outcome was maintenance. Data collection methods included a patient questionnaire, a staff questionnaire and structured interviews with managers.

**Table 1:** **Original and current study definitions of RE-AIM dimensions**

**Dimension**	**Original definitions**	**Current study**		
		**Definition**	**Variable**	**Measurement**
Reach	The absolute number, proportion and representativeness of individuals who are willing to participate in a given initiative	The proportion of patients who receive healthy lifestyle promotion in the last 6 months	Proportion of patients	Patient questionnaire
Effectiveness	The impact of an intervention on important outcomes, including potential negative effects, quality of life, and economic outcomes	Self-reported attitudes and competency among staff regarding healthy lifestyle promotion and the coordinated care model	Proportion of staff	Staff questionnaire
Adoption	The absolute number, proportion, and representativeness of settings and intervention agents who are willing to initiate a program	The proportion of staff who engage in healthy lifestyle promotion practice including referring patients to specialized staff on a daily basis	Proportion of staff	Staff questionnaire
Implementation	At the setting level, implementation refers to the intervention agents’ fidelity to the various elements of an intervention’s protocol	Implementation of the Lifestyle team protocol: Multi-disciplinary structure, team manager, referral procedure, and team meetings	Implementation data	Manager interviews
Maintenance	At the individual level: the long-term effects of a program on outcomes after 6 or more months after the most recent intervention contact.	Reach, effectiveness, adoption and implementation outcomes five years after the Lifestyle teams were implemented.	Reach, effectiveness, adoption and implementation variables and data.	Patient and staff questionnaires
				Manager interviews

### Study intervention

The intervention aimed to coordinate lifestyle promotion within each primary centre. Coordination of practice involved screening for risky behaviours, offering brief advice and, if needed, referring to in-house multidisciplinary lifestyle team members (specialized in lifestyle promotion). A protocol stipulated that centres should implement (1) teams with multidisciplinary structures, (2) team managers, (3) regular team meetings and (4) in-house referral procedures for patients with risky behaviours, e.g. sedentary lifestyle, risky alcohol consumption, poor nutrition or tobacco consumption. Apart from the protocol, each centre could decide how to organize the work. This coordinated care model is referred to here as the Lifestyle team.

### Setting and study groups

The study was performed in Östergötland County, Sweden, which has approximately 440 000 inhabitants. The county council has the administrative responsibility for the publicly financed health care. Ten centres were commissioned in 2009 by the county council to implement Lifestyle teams, with the aim of facilitating knowledge exchange between professions, improving the reach of at-risk patients and standardizing coordinated lifestyle promotion at the centres. All centres were bound by the same financial and budgetary constraints and were comparable regarding size, setting and socioeconomic factors. About 26 700 and 26 000 patients were listed at the intervention and control centres, respectively (according to the county council database, 2011). Randomization of the centres was not feasible as the Lifestyle teams were already in place when data collection commenced.

#### Intervention centres

A best-practice inclusion criterion was applied based on data from the county council. Of ten centres that had been commissioned by the county council to implement Lifestyle teams, three were invited to take part in this project. The three centres had started implementing Lifestyle teams at the time of recruitment, which made them suitable for inclusion. All intervention centres were situated in one urban setting.

#### Control centres

Control centres comparable with the intervention centres in terms of size and setting and within the same county council were selected. None of the control centres had been commissioned to implement Lifestyle teams. Control centres were also situated in one urban setting.

### Participants

#### Patients: reach

Data from a national patient survey were used to measure reach [39]. A random sample of patients who had visited their primary care centre (physician or nurse) was invited to complete the survey. In total, 300 patients per primary care centre were invited: 200 patients who had visited a physician and 100 who had visited a nurse. The inclusion criterion for the current study was age 16 years or older.

#### Staff: effectiveness and adoption

All staff with patient contact at the participating centres, which included physicians, nursing professions, dieticians and behavioural therapists, were invited to complete the staff questionnaire.

#### Managers: implementation

All managers (practice managers and Lifestyle team managers) at the six centres were invited and took part in individual structured telephone interviews.

### Measures

#### Patient questionnaire: reach

Reach was assessed using data from a Swedish national patient survey [39]. Data from one item was used: “Did the physician or other staff discuss [lifestyle behaviour] with you?” The item was repeated for eating habits, physical activity, tobacco and alcohol consumption. There were three response options per lifestyle behaviour: (1) yes, at the current visit; (2) yes, at a visit during the last 6 months; and (3) no. Dichotomized response options were used as the primary outcome; responses (1) and (2) were analysed as patients having received lifestyle promotion. Two items about age and gender were included to investigate responder characteristics.

#### Staff questionnaire: effectiveness and adoption

The questionnaire was generated by the research team, based on a thorough review of the research literature, reviewed by an expert panel and pilot tested among target groups. Items were subsequently modified within the research group to capture the aim of the study and achieve face and content validity. Three items measured responder characteristics: age, gender and profession.

Effectiveness was assessed using self-reported attitudes and competency among staff regarding lifestyle promotion practice. Eight items were used with a four-point response scale (from 1 = strongly agree to 4 = strongly disagree) and the alternative “do not know” (see Table 2 for details of the items).

**Table 2:** **Effectiveness comparison (competency and attitudes): number agreeing with statement/total number of staff and (percentage)**

	**3-year follow-up (2011)**				**5-year follow-up (2013)**			
	**Intervention (** ***n*** **= 77),** ***n/N*** **(%)**	**Control (** ***n*** **= 43),** ***n/N*** **(%)**	***P*** **value** ^**1**^	***P*** **value** ^**2**^ **adjusted by centre**	**Intervention (** ***n*** **= 76),** ***n/N*** **(%)**	**Control (** ***n*** **= 56),** ***n/N*** **(%)**	***P*** **value** ^**1**^	***P*** **value** ^**2**^ **adjusted by centre**
**Self-reported attitude**								
There is a need for a Lifestyle team or similar initiative at my centre	67/73 (92)	30/39 (77)	0.028^a^	0.026	66/71 (93)	34/43 (79)	0.029^a^	0.225
It is important that primary care centres offer support regarding healthy living	69/72 (96)	38/39 (97)	1.000^b^	0.699	71/71 (100)	42/43 (98)	0.377^b^	−^3^
Lifestyle counselling is an efficient method to support patients in behaviour change	70/70 (100)	33/37 (89)	0.013^b^	−^3^	61/64 (95)	39/43 (91)	0.435^b^	0.490
Issues regarding healthy lifestyle promotion are prioritized at my centre	36/69 (52)	7/35 (20)	0.002^a^	<0.001	30/64 (47)	5/36 (14)	0.001^a^	<0.001
**Self-reported competency**								
I have sufficient competency to give patients lifestyle advice	65/73 (89)	38/41 (93)	0.744^b^	<0.001	62/70 (89)	36/42 (86)	0.658^a^	0.687
During a typical consultation I have sufficient time to discuss healthy living with patients	38/73 (52)	15/40 (38)	0.138^a^	0.085	35/70 (50)	17/44 (39)	0.236^a^	0.324
There is sufficient competency (knowledge, skills) at my centre to manage issues regarding healthy lifestyle promotion	69/70 (99)	31/38 (82)	0.003^b^	0.002	71/71 (100)	38/42 (90)	0.017^b^	−^3^
Sometimes it is uncomfortable to bring up healthy living with patients	22/73 (30)	13/40 (33)	0.795^a^	0.760	32/68 (47)	16/44 (36)	0.264	0.154

^1^Significance of difference between intervention and control determined by the χ^2^ test^a^ or the Fisher exact test^b^.

^2^Significance of difference between intervention and control determined by logistic regression using robust standard errors in order to adjust for clustering by centre.

^3^Allocation group with too few numbers in some cells due to complete agreement. Adjusted *P* value cannot be estimated.

Adoption was assessed using two items: (1) “How often do you ask patients about their lifestyle behaviours (physical activity, eating habits, and tobacco or alcohol consumption?” and (2) “How often do you refer patients to staff specialized in lifestyle promotion”. Response options for all items were (1) daily, (2) once/several times a week, (3) once/several times a month, (4) less often, and (5) never. Adoption of lifestyle promotion was defined as daily practice, however weekly practice is also reported.

#### Manager interviews: implementation

A structured interview guide was used based on the Lifestyle team protocol. It included four closed-ended questions representing the content of the protocol: multidisciplinary teams, team managers, regular team meetings and in-house referral procedures. A further eight open-ended questions aimed to explore the degree of implementation regarding the teams (size, professions included and what was discussed at meetings); team development (meaning of the teams, review and dissemination of team goals); and referral procedures (dissemination and use among staff).

### Data collection procedure

#### Patient questionnaire: reach

The national patient survey is distributed biannually to a random sample of patients who have visited their physician or nurse during the month of September in 2011 and 2013. For each centre, 300 invitations are sent by post. Patients can choose to complete the survey on paper or online. For the current study, data for the six participating centres was extracted from the national dataset. Data collection procedures were the same for the 3- and 5-year follow-up. No formal written informed research consent was offered to the participants because the survey was part of a national quality assessment survey. However, the participants were informed about the aim of the survey and that participation was voluntary.

#### Staff questionnaire: effectiveness and adoption

The staff questionnaire was distributed via e-mail in September 2011 and 2013. An e-mail was sent to all clinic-based staff at the six centres including information about the study and explaining that answering the questionnaire meant that formal consent to participate was being given. The mail contained a hyperlink to the questionnaire. Two reminders were sent via e-mail 2 and 3 weeks after the initial e-mail.

#### Manager interviews: implementation

Individual telephone interviews were carried out in October 2011 and 2013. An invitation, accompanied by information about the study aims and confidentiality, were sent via e-mail to all managers. Responding by e-mail to this invitation was taken as written informed consent to participate. In the first invitation, information about the subsequent interview was also given. In 2011, data were recorded by note taking using the interview guide as a score sheet to aid accuracy. In 2013, interviews were audio recorded and transcribed. The same score sheet was used in 2011 and 2013 however. The interviews lasted for about 30 minutes and participants could select a suitable time for the interview. All interviews were carried out by K.T.

### Statistical analyses

Differences between the intervention and control groups on reach and adoption were tested using the χ^2^ test. Differences between intervention and control groups on effectiveness were tested using the χ^2^ test or the Fisher exact test in the case of small sample size. Bonferroni adjustment for multiple end points was applied in the analyses of differences.

The binary outcomes of effectiveness and reach were compared between treatment groups with logistic regression using robust standard errors to take account of clustering effects within each primary health care centre (with the STATA command “cluster”). Interview data were analysed using deductive problem-driven content analysis [40]. The analysis was based on the Lifestyle team protocol whereby data describing (degree of) implementation of the four components of the protocol were identified and synthesized.

### Ethical approval

The study was conducted with the approval of the regional Ethical Review Board in Östergötland, Sweden (DNR: IMH-2009-00335).

## Results

### Participant characteristics

#### Patients

A total of 888 eligible responders were included at the 3-year follow-up and 994 at the 5-year follow-up. Details on gender, age and visit (physician or nurse) are shown in Table 3.

**Table 3:** **Patient sample data: age, gender and type of visit** ^**1**^ **for 2011 and 2013**

	**Response rate,** ***n*** **(%)**					
	**Three year follow-up** ^**2**^			**Five year follow-up** ^**3**^		
	**Intervention**	**Control**	**Total**	**Intervention**	**Control**	**Total**
Gender						
Women	251 (59)	282 (63)	533 (61)	295 (60)	278 (57)	575 (58)
Men	173 (41)	167 (37)	340 (39)	198 (40)	212 (43)	410 (42)
Age						
16–44 years	70 (17)	122 (27)	192 (22)	86 (18)	97 (20)	183 (20)
45–65 years	136 (33)	136 (30)	272 (32)	149 (31)	157 (32)	306 (31)
65–74 years	97 (23)	87 (20)	184 (21)	113 (23)	112 (23)	225 (23)
75+ years	113 (27)	102 (23)	215 (25)	136 (28)	120 (25)	256 (26)
Type of visit						
Physician	276 (64)	307 (67)	583 (66)	347 (70)	350 (70)	697 (70)
Nursing profession	157 (36)	148 (33)	305 (34)	150 (30)	147 (30)	297 (30)

^1^Randomized sample of patients who visited their primary care centre.

^2^2011.

^3^2013.

#### Staff

Response rate at the 3-year follow-up was 77% (*n* = 77) for intervention staff and 65% (*n* = 43) for control centres. At the 5-year follow-up, the figures were 84% (*n* = 76) and 76% (*n* = 56) for the intervention and control centres, respectively. Table 4 shows the responder characteristics in terms of gender, age and profession for the intervention and control centres separately.

**Table 4:** **Responder characteristics for the staff questionnaire for 2011 and 2013: age, gender and profession** ^**1**^

	**Response rate,** ***n*** **(%)**					
	**Three year follow-up** ^**3**^			**Five year follow-up** ^**4**^		
	**Intervention**	**Control**	**Total**	**Intervention**	**Control**	**Total**
Gender						
Women	58 (83)	34 (85)	92 (84)	58 (85)	38 (90)	96 (87)
Men	12 (17)	6 (15)	18 (16)	10 (15)	4 (10)	14 (13)
Age, years						
Mean (SD)	48 (11)	47 (11)	48 (11)	48 (12)	48 (11)	48 (11)
Profession						
Physician	16 (25)	17 (45)	33 (32)	13 (20)	6 (15)	19 (18)
Other^2^	49 (75)	21 (55)	70 (68)	54 (81)	35 (85)	89 (68)

^1^Complete sample of clinic-based staff.

^2^Nursing profession or allied health care.

^3^2011.

^4^2013.

#### Managers

All team and practice managers (*n* = 8) took part in the individual interviews at both follow-ups. At one of the intervention centres, the practice manager and the team coordinator was the same person. All were women with a mean age of 57 years (SD 2 years).

### Reach

Total lifestyle promotion (current visit and visit in the last 6 months) was used as the primary outcome in the analyses. For all lifestyle behaviours combined, a significantly larger proportion of patients at control centres, compared with intervention centres, had received lifestyle promotion at the 3-year follow-up but not at the 5-year follow-up (Table 5).

**Table 5:** **Comparison of reach between intervention and control centres: number and percentage of patients who received lifestyle promotion**

	**Three year follow-up (2011)**				**Five year follow-up (2013)**			
	**Intervention (** ***n*** **= 433),** ***n/N*** **(%)**	**Control (** ***n*** **= 455),** ***n/N*** **(%)**	***P*** **value** ^**1**^	***P*** **value** ^**2**^ **adjusted by centre**	**Intervention (** ***n*** **= 497),** ***n/N*** **(%)**	**Control (** ***n*** **= 497),** ***n/N*** **(%)**	***P*** **value** ^**1**^	***P*** **value** ^**2**^ **adjusted by centre**
**Eating habits**								
Current visit	54/411 (13)	63/439 (14)	0.608	0.620	55/485 (11)	50/481 (10)	0.637	0.346
Last 6 months	41/411 (10)	53/439 (12)	0.330	0.510	34/485 (7)	71/481 (15)	<0.001	<0.001
Total^2^	95/411 (23)	116/439 (26)	0.264	0.398	89/485 (18)	121/481 (25)	0.010	0.003
**Physical activity**								
Current visit	71/403 (18)	79/433 (18)	0.813	0.846	72/482 (15)	91/478 (19)	0.091	0.142
Last 6 months	46/403 (11)	76/433 (18)	0.012	<0.001	53/482 (11)	81/478 (17)	0.008	0.003
Total^3^	117/403 (29)	155/433 (36)	0.037	0.066	125/482 (26)	172/478 (36)	0.001	0.035
**Tobacco consumption**								
Current visit	70/402 (17)	82/428 (19)	0.516	0.441	54/482 (11)	74/477 (16)	0.050	0.083
Last 6 months	39/402 (10)	39/428 (9)	0.771	0.757	35/482 (7)	47/477 (10)	0.151	0.061
Total^3^	109/402 (27)	121/428 (28)	0.710	0.549	89/482 (18)	121/477 (25)	0.010	0.068
**Alcohol consumption**								
Current visit	49/406 (12)	48/432 (11)	0.665	0.463	33/480 (7)	56/476 (12)	0.009	0.037
Last 6 months	30/406 (7)	36/432 (8)	0.612	0.484	23/480 (5)	48/476 (10)	0.002	<0.001
Total^3^	79/406 (19)	84/432 (19)	0.996	0.994	56/480 (12)	104/476 (22)	<0.001	0.003
**Lifestyles combined**								
Current visit	110/416 (26)	140/441 (32)	0.088	0.170	113/488 (23)	126/485 (26)	0.306	0.487
Last 6 months	74/416 (18)	101/441 (23)	0.063	0.081	82/488 (17)	118/485 (24)	0.004	0.006
Total^3^	169/416 (41)	211/441 (48)	0.033	0.028	177/488 (36)	211/485 (44)	0.021	0.198

^1^Significance of difference between intervention and control determined by the χ^2^ test.

^2^Significance of difference between intervention and control determined by logistic regression using standard robust errors in order to adjust for clustering by centre.

^3^Current visit and visit in last 6 months combined.

At the 3-year follow-up, significant differences remained only for physical activity promotion when analysing lifestyle behaviours separately adjusted for a clustering effect of centres. However, the figures for the 5-year follow-up show that patients at the control centres significantly more often received lifestyle promotion for all lifestyle behaviours except for advice about smoking. The reach for separate and combined lifestyle behaviours at the 3- and 5-year follow-ups is compared in Table 5.

### Effectiveness

Significant differences were found between the intervention and control centres for five of the eight competency and attitude items at the 3-year follow-up and for two items at the 5-year follow-up (Table 5). At both the 3- and the 5-year follow-up, intervention staff were significantly more likely to agree that issues regarding healthy lifestyle promotion are prioritized at their centre and that there is sufficient competency (knowledge, skills) at their centre to manage lifestyle promotion. The effectiveness measurements for the intervention and control centres at the 3- and 5-year follow-ups are compared in Table 2.

### Adoption

No significant differences were found between intervention and control centres at either follow-up regarding daily lifestyle promotion practice. At the 3-year follow-up, 47% (*n* = 34) of intervention staff and 59% (*n* = 24) of the control staff reported that they asked patients about their lifestyles on a daily basis. At the 5-year follow-up, these figures were 36% (*n* = 26) and 45% (*n* = 21) for intervention and control centres, respectively. Both intervention and control staff referred patients to other professions who specialized in lifestyle promotion. At the 3-year follow-up, 27% (*n* = 20) of intervention staff and 31% (*n* = 13) of control staff did this on a weekly basis. The same figures for the 5-year follow-up were 25% (*n* = 18) for intervention staff and 21% (*n* = 10) for control staff. Only one to three staff members reported that they referred patients daily. This was true for both intervention and control centres and for both time points.

### Implementation

In terms of the original protocol, the Lifestyle teams were implemented at the intervention centres in 2011 and were still in place in 2013. All three intervention centres had implemented multidisciplinary Lifestyle team structures, team managers, regular team meetings and referral procedures. Team size varied between 2011 and 2013: team A (6–7 members), team B (10–15 members) and team C (10–11 members). Most of the team members were women but all teams had 1 or 2 male members. Professions included in the teams were behavioural therapists, dieticians, district nurses, specialized nurses and practice managers. Two teams also included physicians (A and B) and medical secretaries (B and C). Team meetings provided the opportunity to share knowledge and discuss current events and project improvements. Concrete topics such as theme days and task delegation were also discussed. With regard to the degree of implementation, structures for the referral procedures were put in place but used inconsistently among staff. The Lifestyle teams were reported to be the vehicle for driving lifestyle promotion work forward and an important forum for knowledge exchange.

## Discussion

This study aimed to evaluate the long-term impact of a multidisciplinary team approach to coordinated lifestyle promotion in primary care. The findings were consistent over time regarding all outcomes. The reach of patients or adoption among staff did not occur at a higher rate in the intervention centres compared with the control centres at either the 3- or 5-year follow-up. However, intervention staff were initially more positive on most of the effectiveness outcomes, but only two of the eight items remained significant at the 5-year follow-up. Not surprisingly, the two significant items were that healthy lifestyle promotion is prioritized and that there is sufficient competency at the centre regarding healthy lifestyle promotion. The intervention was implemented consistently with the original protocol, which included structural opportunities for coordinated care. However, referral procedures for lifestyle promotion were used inconsistently by the staff.

### Maintenance

#### Lifestyle promotion practice

Lifestyle promotion practice was measured both from a patient and a staff perspective. The findings show that the Lifestyle teams had limited impact on the rate of lifestyle promotion when compared with the control centres. However, the intervention and control groups were comparable regarding various factors. The determinants of lifestyle promotion are multifaceted. Variations in practice rates can be explained by intrapersonal (e.g. motivation); interpersonal (e.g. patient response) and organizational (e.g. resources) factors [11,23-27]. It is unclear from our study how these factors may have interacted and facilitated lifestyle promotion practice at the control centres.

One explanation of the findings could be how the intervention was used and to what degree referral routines were implemented. The findings show that structures for coordinated care were implemented but the utility of these structures was inconsistent. This may have compromised the capacity of the intervention to reach patients (especially the capacity among specialized staff). Thus, the degree of implementation may have limited the intervention and influenced the evaluation in this study [41,42]. Considering the many factors influencing clinical practice, the Lifestyle teams may have offered organizational opportunities in terms of resources for referral but the findings suggest that the teams had limited impact on interpersonal factors such as referral behaviour.

A potential methodological explanation for the findings may be the crude measurement of lifestyle promotion that was used in this study. All types of lifestyle promotion (from screening to extended sessions) were included, which may also explain the rather high rates that were found. At least about 25% of patients received lifestyle promotion at both the intervention and control centres. These figures are high compared with previous studies that report around 10% of patients receive lifestyle advice [17]. By not taking into account the different facets of lifestyle promotion such as content, duration, patient satisfaction or even patient clinical outcomes, our study may not have captured a complete picture of lifestyle promotion practice at these centres. These aspects could be included in future studies.

#### Attitudes and competency

Conceptual differences between intervention and control staff diminished at the 5-year follow-up. Thus, only one attitude and one competency item had significantly higher agreement in intervention centres compared with control centres: how lifestyle promotion was prioritized at the centre and that there was sufficient competency concerning healthy lifestyle promotion. Considering that these differences were resilient over time, they suggest that the Lifestyle teams had an impact on these aspects. Similarly, interview data showed that the Lifestyle teams had an important role at centre level, driving lifestyle promotion work forward. Thus, the Lifestyle teams may have offered concrete opportunities for lifestyle promotion practice (e.g. referral resources) and contributed to supporting conditions at centre level (e.g. shared sense of competency). Although the findings suggest that the Lifestyle teams reduced institutional barriers, the teams did not have any additional impact on practice behaviour when compared with the control centres. Thus, a Lifestyle team or similar intervention would have to be combined with interventions aimed at targeting inter-relational barriers of practice. There is increasing support that multifaceted interventions, tailored to specific barriers, are the most effective approach to achieve practice change [43].

#### Implementation

The Lifestyle teams were implemented in terms of the content of the original protocol. However, the interview data suggested that staff used the referral procedures inconsistently. Research has shown that implementing structural components does not necessarily result in coordinated care, which requires commitment from all staff groups [44]. As previously mentioned, the suboptimal degree of implementation may have influenced the findings and subsequent evaluation [41,42]. The study confirms the importance of including the degree of implementation in intervention studies. Furthermore, the findings highlight the importance of defining strategies on how to engage staff in referral when designing coordinated care interventions.

### Methodological considerations

The study did not include pretest data on lifestyle promotion due to logistical constraints relating to the timing of the evaluation. Furthermore, randomization of the centres was not feasible due to the commissioning of the intervention. We tried to deal with these issues by comparing intervention centres with control centres that were comparable regarding lifestyle promotion guidelines, financial and budgetary constraints, size, setting and socioeconomic factors. Furthermore, no established questionnaires were found to measure the variables. The research group searched for suitable validated instruments to measure reach, effectiveness and adoption with little success. The items were tailored to fit the local context and were piloted before use to achieve face validity. The study focused on outcomes at the staff level. For a comprehensive understanding of the impact of the Lifestyle teams, and similar coordination interventions, future studies could consider outcomes at the patient level.

## Conclusions

The findings were consistent over time. Intervention centres did not show higher rates on reach of patients or adoption among staff at the 3- or 5-year follow-ups. Some conceptual differences between intervention and control staff remained over time in that the intervention staff were more positive about one attitude and one competency statement compared with control staff. The Lifestyle team protocol was implemented at all intervention centres, and included structural opportunities for coordinated care. Lifestyle teams were perceived to have an important role at the centres in driving the lifestyle promotion work forward and being a forum for knowledge exchange. However, resources to refer patients to specialized staff were used inconsistently by the staff. The Lifestyle teams may have offered opportunities for lifestyle promotion practice and contributed to enabling conditions at centre level but had limited impact on the rate of lifestyle promotion practice.
